# PyMICE - a Python™ library for analysis of mice behaviour

**DOI:** 10.1186/1471-2202-16-S1-P145

**Published:** 2015-12-18

**Authors:** Jakub M Kowalski, Alicja Puścian, Zofia Mijakowska, Maria Nalberczak, Kasia Radwañska, Szymon Łęski

**Affiliations:** 1 Department of Neurophysiology, Nencki Institute of Experimental Biology, Warsaw, 02-093, Poland; 2 Department of Molecular and Cellular Neurobiology, Nencki Institute of Experimental Biology, Warsaw, 02-093, Poland

## 

Manual analysis of abundant behavioral data produced by automated systems for long-time monitoring of a group of animals is extremely inefficient and error prone. Some systems (like IntelliCage™) are shipped with software enough for basic analysis of the data, however lacking flexibility for more advanced analyses.

To facilitate research reproducibility, same analysis of same data should always yield same results. One (possibly the best) way to achieve such robustness is to have the process automated (due to the IT slogan *"let the computer do the work"*), therefore reducing number of possible human errors. The other advantage of such approach is that modern computers are both faster and more precise than humans when dealing with numbers.

**Figure 1:**
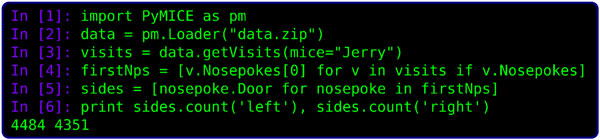
**A six-line example of data analysis with PyMICE library**. Firstly the library is loaded, secondly data are read from file "data.zip". In the third line a list of all visits of mouse named "Jerry" is obtained. Then the first nose poke (if any) from every visit is selected and then - the side of the nose poked door. Finally numbers of left and right doors nose poked as first are counted.

An automated analysis workflow can be created easily if there is a possibility of a convenient access to data. That is the purpose of development of PyMICE library by our laboratory. The library provides its user with an object oriented application programming interface (API) and a data abstraction layer - therefore shifting ones focus from the form the data is provided to the data itself. A simple analysis can be performed in just a few lines of readable source code (see Figure 1 for an example). Moreover, the library comes with auxiliary tools supporting development of analysis workflows. Some of them facilitate data validation while other are dedicated for workflow configuration.

